# *IDH1*: Linking Metabolism and Epigenetics

**DOI:** 10.3389/fgene.2018.00493

**Published:** 2018-10-23

**Authors:** Silvia Raineri, Jane Mellor

**Affiliations:** ^1^Department of Biochemistry, University of Oxford, Oxford, United Kingdom; ^2^Chronos Therapeutics, Oxford, United Kingdom

**Keywords:** IDH1 mutation, 2HG, TADs, hypermethylation, glioma, metabolism, epigenetics

## Abstract

Mutations in genes encoding enzymes of the tricarboxylic acid cycle often contribute to cancer development and progression by disrupting cell metabolism and altering the epigenetic landscape. This is exemplified by the isoforms of isocitrate dehydrogenase (IDH1/2), which metabolize isocitrate to α-Ketoglutarate (α-KG). Gain of function mutations in *IDH1* or *IDH2* result in reduced levels of α-KG as a result of increased formation of D-2-Hydroxyglutarate (2-HG). α-KG is an essential co-factor for certain histone and DNA demethylases, while 2-HG is a competitive inhibitor. These *IDH1/2* mutations are thought to result in hypermethylated histones and DNA which in turn alters gene expression and drives cancer progression. While this model seems to be generally accepted in the field, the exact molecular mechanisms still remain elusive. How much of this model has been rigorously demonstrated and what is just being assumed? Are the effects genome-wide or focused on specific loci? This *Perspective* aims at elucidating the key questions that remain to be addressed, the experimental techniques that could be used to gain further insight into the molecular mechanisms involved and the additional consequences of these mutations beyond DNA and protein methylation.

## Introduction

Metabolism and epigenetics are highly interconnected. Several proteins involved in metabolic pathways also participate to chromatin remodeling and gene regulation by producing co-factors or substrates used by epigenetic writers (Wellen et al., 2009). One such example is isocitrate dehydrogenase (IDH). IDH enzymes metabolize Isocitrate to α-Ketoglutarate, either in the mitochondrion as a step of the Krebs cycle (IDH2) or in the cytoplasm (IDH1) (M Gagné et al., 2017). α-Ketoglutarate produced by this reaction serves as a co-factor for several α-Ketoglutarate-dependent dioxygenases, notably the ten-eleven translocation (TET) family of DNA demethylases and the Jumonji (Jmj) family of histone demethylases (Tsukada et al., 2006; Tahiliani et al., 2009).

Interestingly, *IDH* mutations are common in several types of cancers, including ∼80% of glioblastomas, ∼40% chondrosarcomas, 20% of acute myeloid leukemias (AML), ∼55% sinonasal undifferentiated carcinoma, and 1% prostate cancer (Table 1; Amary et al., 2011; Pansuriya et al., 2011; Liu et al., 2013; Adam et al., 2014; Abeshouse et al., 2015). These heterozygous mutations can be found in substrate binding residues of both IDH1 (R132H) and IDH2 (R140Q, R172K) (Yan et al., 2009). While IDH1 mutations are more common in gliomas (80%) and AML (20%), IDH2 mutations occur more frequently in AML (20%) and cholangiosarcomas (20%) (Mondesir et al., 2016).

**Table 1:** Frequencies of IDH1/2 mutations in different types of cancer.

Cancer type	IDH mutant	Frequency (%)	Reference
Glioma	IDH1 and IDH2	80	Yan et al., 2009
AML	IDH1 and IDH2	20	Mondesir et al., 2016
Prostate	IDH1	1	Abeshouse et al., 2015
Angioimmunoblastic T-cell lymphoma	IDH2	20	Cairns et al., 2012
Cholangiocarcinoma	IDH1 and IDH2	23	Borger et al., 2012
Chondrosarcoma	IDH1 and IDH2	56	Amary et al., 2011
Ollier disease	IDH1 and IDH2	81	Pansuriya et al., 2011
Maffucci syndrome	IDH1 and IDH2	77	Pansuriya et al., 2011
Thyroid cancer	IDH1	11	Murugan et al., 2017
Sinonasal undifferentiated carcinoma	IDH2	55	Dogan et al., 2017; Jo et al., 2017; Mito et al., 2018

Both mutations have been associated with a relatively better prognosis and induce a gain of function that causes further processing of α-Ketoglutarate into 2-hydroxyglutarate (2HG) (Dang et al., 2009), an oncometabolite linked with tumor progression (Weller et al., 2011). Given the large number of studies on these mutations and their impact on cancer progression, several targeted inhibitors of the mutant form of IDH1 or IDH2 have been developed and have now reached the clinical trial stage (Table 2; DiNardo et al., 2016; Popovici-Muller et al., 2018; Yen et al., 2017, 2018). Despite the many similarities between mutations in IDH1 and IDH2, in this *Perspective* the focus will primarily be on IDH1.

**Table 2:** Current targeted therapies for IDH1/2-mutant tumors.

Drug	Target	Effect	Clinical stage	Clinical trial ID	Predicted impact on methylation
AG-221	Mutant IDH2	Suppression of 2HG production, induction of cell differentiation	Phase 1/2	NCT01915498	Restoration of DNA and histone demethylases activity. Methylation levels back to their original state. CTCF binding restored.
AG-120	Mutant IDH1	Suppression of 2HG production	Phase 1	NCT02074839	
IDH305	Mutant IDH1	Suppression of 2HG production and cell proliferation	Phase 1	NCT02381886	
AG-881	Mutant IDH1 and 2	Suppression of 2HG production, induction of cell differentiation	Phase 1	NCT02492737	

**FIGURE 1:**
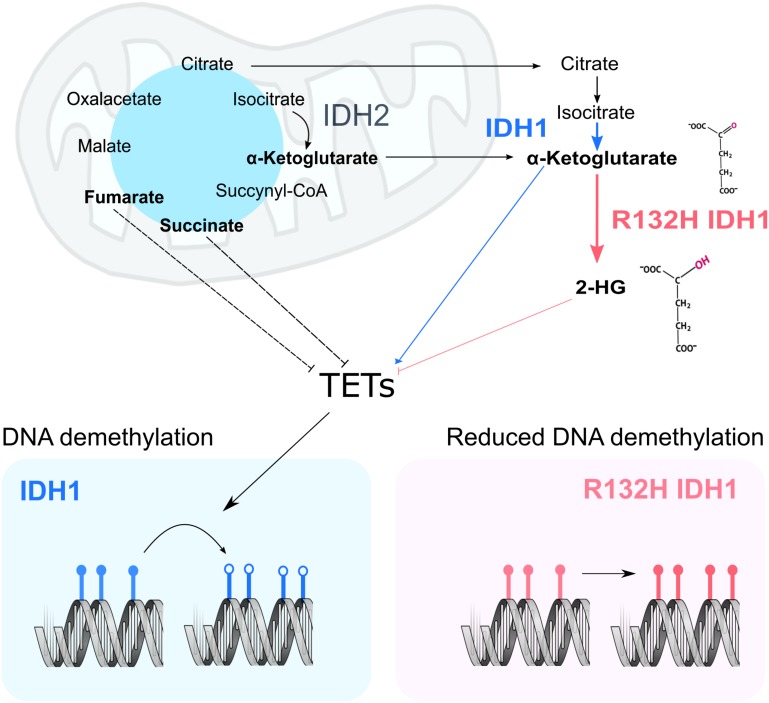
Current model of the impact of IDH1 mutation on chromatin remodeling. In a WT cell, IDH1/2 (blue and black) metabolize Isocitrate into α-Ketoglutarate. Upon mutation of IDH1 (pink panel), α-Ketoglutarate is processed into 2HG. In other cancer settings, mutations in fumarate hydratase and succinate dehydrogenase result in the accumulation of fumarate and succinate, which may inhibit TETs. α-Ketoglutarate acts as co-factor in the nucleus for both DNA demethylases (TETs) and histone demethylases (KDMs). Their activity ensures the correct levels of DNA (bottom panel) and protein methylation in the cell. More specifically, TETs act on methylated DNA sequences (filled lollipops), starting a reaction chain that will ultimately lead to methyl group removal (empty lollipops).

According to the current model, the structural similarity between 2HG and α-Ketoglutarate causes inhibition of both histone and DNA demethylases, inducing an increased methylated state in the nucleus which leads to gene expression deregulation and promotes cancer development (Xu et al., 2011; Figure 1). This inhibition is also achieved upon mutations in other enzymes of the tricarboxylic acid cycle, mainly *Fumarate hydratase* (*FH*) and *Succinate dehydrogenase* (*SDH*) (Xiao et al., 2012). These losses of function mutations induce excessive accumulation of their respective substrates, Fumarate and Succinate, which then act as competitive inhibitors of α-Ketoglutarate-dependent dioxygenases. This model suggests a metabolic basis for the changes observed in chromatin as a result of the *IDH1* mutations. Despite a growing body of evidence, the exact molecular mechanism and consequences of 2HG production are still largely unknown. This *Perspective* aims to discuss the current idea about the effect of the *IDH1* mutations on the chromatin structure, reflect upon the proposed model, and identify current weaknesses and key questions that still need to be addressed.

## Adding a New Piece to the Puzzle: The Consequence of the *IDH1* Mutation on the Formation of Chromatin Domains

**FIGURE 2:**
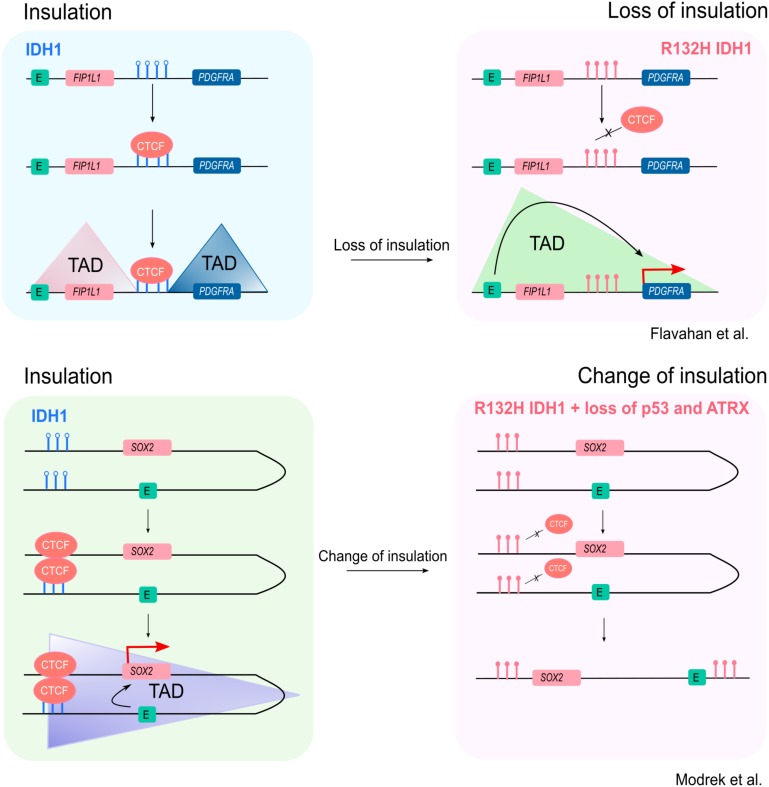
Changes in insulation of TADs as a consequence of IDH1 mutation. According to the model suggested by Flavahan et al. (2016, top panel) in a WT IDH1 setting, CTCF is able to bind to particular target sites along the genome, in certain contexts acting as a functional insulator, creating DNA domains that separate neighboring regions. At CTCF binding sites containing the CG dinucleotide, changes in levels of DNA methylation influence its DNA binding. Specifically, the production of 2HG inhibits TETs, leading to hypermethylation (red, filled lollipops) of CTCF-binding sites, reducing binding (right panel). In the context of PDGFRA, the formation of domains sequesters an enhancer upstream the FIP1L1 gene into a separate domain (upper left panel). Upon IDH1 mutation, however, the overall increase in methylation levels due production of 2HG induces loss of CTCF binding to its target sites, leading to loss of insulation between TADs. In this example, destruction of a boundary induces a rearrangement that brings PDGFRA in proximity of an enhancer found upstream the FIP1L1 gene, thus inducing its deregulation (upper right panel). The example described in Modrek et al. (2017) (lower panel) focuses on the *SOX2* locus. Here, in the WT IDH1 context, CTCF binding induces the formation of a loop that brings the *SOX2* gene and its promoter in close proximity to a downstream enhancer that would be otherwise out of reach (lower left panel). This interaction favors *SOX2* expression. Upon a three-hit mechanism that includes mutation of *IDH1* and loss of both *p53* and *ATRX*, reduced binding of CTCF at the *SOX2* locus impairs the formation of the loop, thus causing a change, rather than a loss, of insulation. In this new setting, the downstream enhancer is too far away to interact with the gene, whose expression is now downregulated (lower right panel). Filled lollipops = methylated DNA; empty lollipops = unmethylated DNA.

Recently, Flavahan et al. (2016) added a further step in the model by focusing on chromatin domains. The genome is organized into self-interacting genomic regions, called topologically associated domains (TADs) (Bickmore and Van Steensel, 2013). Proteins like the CCCTC-binding factor (CTCF) often act as insulators (Nakahashi et al., 2013; Hanssen et al., 2017), separating TADs from one another by binding to sequence-specific sites on the DNA (Dixon et al., 2012). This can effectively insulate a gene on one domain from activation by an exogenous enhancer on a neighboring domain. By studying the 3D DNA structure in cells or patient-derived samples bearing the most common *IDH1* mutation, R132H, the group reports that high DNA methylation levels might prevent binding of insulator proteins to the DNA, thus destroying existing chromatin domains and promoting the formation of new TADs within the chromatin. This mechanism contributes to the dysregulation of an already compromised gene expression. Indeed, they propose that loss of a domain boundary between a constitutive enhancer upstream of the *FIP1L1* gene and the gene encoding PDGF receptor alpha (PDGFRA) induces its aberrant expression (Flavahan et al., 2016; Figure 2).

## Deconstructing the Model

The current model is defined by three main steps. First, mutant *IDH1* produces 2HG from α-Ketoglutarate. Second, 2HG inhibits histone and DNA demethylases, causing an increase in methylation levels. Finally, methylation on CTCF binding sites in the DNA inhibits CTCF binding and induces rearrangement of TADs.

The second step in this model presents complications when considering its impact on gene expression, as the specific effects of increased global hypermethylation are context-dependent. For example, increased promoter DNA methylation (potentially repressive) may have different consequences from increased insulator methylation. Flavahan et al. (2016) report a five-fold increase of *PDGFRA* expression in *R132H IDH1* glioma cells, which they claim is due to the loss of insulation leading to the new interaction between a strong enhancer upstream of the *FIP1L1* gene and the *PDGFRA* promoter. However, given that mutation of *IDH1* is sufficient to induce a CpG island methylator phenotype (G-CIMP) (Turcan et al., 2012) and that the UCSC Genome Browser describes the presence of a CpG island within the *PDGFRA* promoter, an expectation is that in the *R132H IDH1* context, this promoter becomes hypermethylated, which is generally linked to transcriptional inactivation (De Smet et al., 1999). There are two possible explanations for this apparent contradiction. First, the proximity of a stronger enhancer is able to overcome what would be an otherwise silencing effect on gene expression. Second, the promoter escapes DNA hypermethylation due to H3K4me3, a histone modification associated with active genes (Santos-Rosa et al., 2002). Given its mutual exclusivity with DNA methylation (Weber et al., 2007), the presence of this mark could protect DNA sequences from being methylated by hindering binding of Dnmt3L, a protein thought to help the *de novo* methyltransferase Dnmt3A/B (Hata et al., 2002; Ooi et al., 2007). Interestingly, H3K4me3 is one of the few methylation marks that does not seem to increase upon *IDH1* mutation (Lu et al., 2012). Potentially, it could mean that those DNA sequences associated with H3K4me3 remain unmethylated, and transcriptionally active, in spite of the global increase in methylation.

The third step in this model, that methylation on CTCF binding sites inhibits CTCF binding to induce domain rearrangements, is the most challenging to validate. The first indications that 2HG-driven hypermethylation induces a rearrangement of chromatin domains was reported in Flavahan et al. (2016). The authors exploit a combination of computational methods, chromatin immunoprecipitation followed by sequencing (ChIP-Seq) to assess where on the DNA CTCF interacts, and publicly available data reporting changes to degrees of DNA methylation in glioma cells with or without the *IDH1* mutation (Noushmehr et al., 2010). Results suggest that tumors bearing the mutated *IDH1* lose CTCF binding and show increased DNA methylation. Next, the authors proceed to analyzing higher order chromatin structures.

Chromatin capture sequencing techniques are based on the crosslinking of cells with formaldehyde to link together chromatin segments in close spatial proximity. Chromatin is then digested with restriction enzymes and ligated in DNA hybrid molecules containing parts of the two sequences of DNA that were interacting with each other. In the classical chromosome conformation capture (3C) protocol, a real time PCR is performed using specific primers to amplify a locus of interest. In more advanced methods, such as Hi-C, gaps in the DNA sequences are filled in with biotinylated nucleotides after restriction enzyme digestion. Hybrid sequences are then pulled down and used to prepare libraries, enabling whole-genome analysis of the interactions, as opposed to one locus at a time (Dekker et al., 2013).

Flavahan et al. (2016) couple publicly available data from Hi-C, used to assess chromatin domains genome wide (Rao et al., 2014), and RNA sequencing experiments (Verhaak et al., 2010; Brennan et al., 2013) to compute the correlation of gene expression between genes contained in the same chromatin domain and those belonging to neighboring domains. When comparing gene expression from gliomas with or without the *IDH* mutation, they find that in mutated gliomas, genes tend to correlate better with genes from a close, but separate, domain than with genes within their same domain. Finally, they scan these loci of interest for genes whose expression is higher in *IDH1* mutant gliomas, obtaining a final list of genes. As a result of this key computational experiment, the authors select the locus on chromosome 4 between *FIP1L1* and *PDGFRA* as an example of disrupted insulation. To validate their hypothesis, the authors use a series of 3C experiments around the *FIP1L1-PDGFRA* locus to show the altered domain structure.

The way they formulated their hypothesis using a largely computational method raises an interesting problem. While the publicly available RNA sequencing datasets used derive from glioma samples, the Hi-C experiments had been carried out in a series of very different cell lines: IMR90 human lung fibroblasts, GM12878 lymphoblastoid cells, K562 bone marrow chronic myelogenous leukemia cells and NHEK normal epidermal keratinocytes. Publicly available datasets are a valuable resource to test out a hypothesis, especially given the complexity of carrying out a Hi-C experiment, and it could be argued that if the same conformations exist in different cell types they are likely to be widely conserved. On the other hand, is it valid to use 3C data from different cell types to make specific assumptions about glioma cells, when they might have a completely different arrangement of TADs? To address this, the authors follow their computational analysis with local 3C experiments to assess the interactions at the *FIP1L1-PDGFRA* locus in a series of primary glioma cells and a panel of glioblastoma cell lines. To confirm that loss of insulation, and the subsequent *PDGFRA* overexpression, is indeed due to loss of CTCF binding, Flavahan et al. (2016) genetically edited out the putative CTCF binding site between *FIP1L1* and *PDGFRA* using the CRISPR-Cas9 system. However, instead of measuring the actual formation of a novel interaction through a 3C experiment on such CRISPR-modified cells, their readout is based on *PDGFRA* expression levels obtained by qPCR, and the presence of PDGFRα on the cell membrane, measured by FACS. Thus, they do not actually show that the CTCF and methylation-dependent function of the insulator alters higher order chromatin loops.

## From Local to Genome-Wide Mechanism: Challenges Ahead

Recently, a second example of higher order chromatin structure alteration following mutation of *IDH1* has been published. In their paper, Modrek et al. (2017) use a combination of R132H *IDH1* and silencing of both *p53* and *ATRX* to model lower-grade glioma genetic lesions in human neural stem cells (HNSC). They propose that reduced CTCF binding around the *SOX2* gene is associated with loss of a DNA domain or loop which normally positions the *SOX2* promoter in close proximity to an enhancer, ∼0.5–1 Mb downstream from *SOX2*. Thus, altered CTCF binding is associated with downregulation of *SOX2* expression, blocking differentiation, in contrast to the increase expression of *PDGFRA* in the Flavahan study.

Despite findings with similarities to the model proposed by Flavahan et al. (2016), the Modrek et al. (2017) paper offers some interesting points for discussion. When looking at the methylation levels around the *SOX2* promoter, there were no striking differences between their three-hit cells and the empty-vector controls. Only when taking into account a much larger region around *SOX2* (1.2 Mb) they were able to identify specific areas up- and down-stream of the gene that indeed showed increased methylation levels. When comparing these areas to the CTCF ChIP-Seq data from Flavahan et al. (2016), they identified five potential CTCF binding sites that could be influenced by increased DNA methylation. The authors face what will be the ultimate challenge for future research in the field: how to correctly map the domain boundaries by merging the CTCF binding data with the DNA methylation and the chromatin conformation information and to show whether one or all five of these CTCF binding sites contribute to a chromatin conformation that facilitates promoter:enhancer interactions. Indeed, this will require the combination of a solid mapping of CTCF binding sites across the genome, a reliable description of the chromatin domains in both *IDH1* wild-type and mutated cells, a thorough annotation of the H3K27ac mark to define enhancer sequences, an accurate portrayal of DNA methylation landscape and finally a method to validate the findings.

Matching different -omics into a single picture of the epigenetic state of the cell will prove to be difficult. When starting from publicly available data, the main issue will be choosing the appropriate datasets. Data from the different -omics might not be available in the same cell line, or, at times, even the same cell type. Furthermore, sequences might have been analyzed or normalized according to different methods, and raw data is not always disclosed. All these might seem small details, but they add up introducing biases in the analysis, making it difficult for the scientist to draw clear conclusions.

A second option is carrying out the -omics experiments in the lab. This would ensure consistency of the cellular model, and a better control over the technical biases that might be introduced in the experimental procedure. However, this would require designing a proper cellular model. Many papers have generated their own system by stably transfecting an empty vector, wild-type *IDH1* or R132H *IDH1* into glioma cell lines. While this might have been a good solution to initially study the alterations induced by the *IDH1* R132H mutation, if the focus has now shifted to higher order chromatin structure then perhaps introducing a gene via transfection might cause some alterations to the DNA loops by itself. A possibility could be to selectively introduce the *IDH1* R132H mutation in a wild-type *IDH1* cell line using the CRISPR-Cas9 system.

Obtaining the data will only solve part of the problem, as potential difficulties will lie in correctly mapping the TADs and understanding which CTCF binding sites are responsible for the disruption or the formation of new contacts between gene promoters and enhancers. Multiomics is an approach to data analysis that aims at integrating, rather than comparing, results from different -omics experiments, in an effort to model complex phenotypes. Despite this being a task that presents its own challenges, it could be the most appropriate way to move forward. The final challenge will be to define whether *IDH1* mutations affect particularly sensitive loci containing potential oncogenes such as *PDGFRA* and *SOX2*, or whether this is a genome-wide mechanism.

## Future Directions: the Role of Histones

Another question that needs to be addressed is whether histone hypermethylation plays any role in remodeling higher order chromatin structures. 2HG production induces inhibition of Jumonji-C domain histone demethylases (Xu et al., 2011) (KDMs), with a corresponding increase in selective methylation marks, including H3K27me3 (Lu et al., 2012). However, whether this increase in histone methylation affects formation of higher order chromatin structures is unknown.

Studies in *Drosophila* have described how H3K27me3 distribution seems to divide the genome into H3K27me3-enriched areas, corresponding to prominent TADs domains and delimited by CTCF binding sites, or H3K27me3–depleted areas, whose distribution correlates with TADs boundaries (Van Bortle et al., 2012; El-Sharnouby et al., 2017). While the connection between H3K27me3 and CTCF in maintaining domains is generally accepted, how CTCF exerts its insulator function is unknown. Knockdown of CTCF has been reported to have different outcomes on H3K27me3 distribution: at the genome-wide level, it does not cause spreading of this epigenetic mark into neighboring domains (Schwartz et al., 2012; Van Bortle et al., 2012). Paradoxically, when considering single genes, “spill-over” of the H3K27me3 chromatin mark into the flanking regions is reported in CTCF knock-downs (Soto-Reyes and Recillas-Targa, 2010; Essafi et al., 2011). The next steps in the field will be fundamental to help clarifying these discordant results, perhaps by focusing on few specific CTCF binding sites to delete with the CRISPR-Cas9 technology, followed by assessment of H3K27me3 levels, rather than aiming at a global CTCF knock-down.

Future research could build upon these studies by investigating the levels of histone methylation in an *IDH1* mutant setting and assessing whether the mutation has any impact on DNA domain formation.

## Conclusion

IDH1 production of α-Ketoglutarate fuels the activity of several proteins, including DNA and histone demethylases. This effect is impaired upon mutation of the *IDH1* gene, when the further processing of α-Ketoglutarate to 2HG inhibits both DNA and histone demethylases, thus increasing the methylation level within the cell, with disruptive effects on gene expression and cell differentiation. This phenomenon has been observed in different types of cancer, but more consistently in around 80% of glioblastomas and 20% of AMLs. Thus, defining the molecular consequences of this mutation and the different cellular processes affected could provide new druggable targets for efficient therapy, or help in finding predictive biomarkers.

Research in the field has made an important progress over the past few years, after the discovery that *IDH1* mutations might also induce alterations in the 3D DNA structure. However, these recent results also highlight new challenges. On the experimental side, there is currently a lack of proper cellular models in which to introduce (or rescue) the R132H *IDH1* mutation without the risk of perturbing the DNA loops. It will be interesting to see whether cutting edge genome editing techniques will help in designing an adequate model. On the computational side, the multiomics approach of integrating different -omics into one comprehensive mapping of insulator binding sites, enhancer-associated chromatin marks and methylation patterns is required before attempting to find which interactions are lost and which are newly formed upon *IDH1* mutation.

## Author Contributions

SR wrote the manuscript and designed the figures. JM critically reviewed and edited the manuscript.

## Conflict of Interest Statement

JM acts as an advisor to and holds stock in Oxford BioDynamics Plc., Chronos Therapeutics Ltd., and Sibelius Natural Products Ltd. SR is employed by Chronos Therapeutics Ltd.

## References

[B1] Abeshouse A. Ahn J. Akbani R. Ally A. Amin S. Andry C. D. (2015). The molecular taxonomy of primary prostate cancer. *Cell* 163 1011–1025. 10.1016/J.CELL.2015.10.025 26544944 PMC4695400

[B2] Adam J. Yang M. Soga T. Pollard P. J. (2014). Rare insights into cancer biology. *Oncogene* 33 2547–2556. 10.1038/onc.2013.222 23812428

[B3] Amary M. F. Bacsi K. Maggiani F. Damato S. Halai D. Berisha F. (2011). IDH1 and IDH2 mutations are frequent events in central chondrosarcoma and central and periosteal chondromas but not in other mesenchymal tumours. *J. Pathol.* 224 334–343. 10.1002/path.2913 21598255

[B4] Bickmore W. A. Van Steensel B. (2013). Genome architecture: domain organization of interphase chromosomes. *Cell* 152 1270–1284. 10.1016/j.cell.2013.02.001 23498936

[B5] Borger D. R. Tanabe K. K. Fan K. C. Lopez H. U. Fantin V. R. Straley K. S. (2012). Frequent mutation of Isocitrate Dehydrogenase (IDH)1 and IDH2 in Cholangiocarcinoma identified through broad-based tumor genotyping. *Oncologist* 17 72–79. 10.1634/theoncologist.2011-0386 22180306 PMC3267826

[B6] Brennan C. W. Verhaak R. G. W. Mckenna A. Campos B. Noushmehr H. Salama S. R. (2013). The somatic genomic landscape of glioblastoma. *Cell* 30 462–462. 10.1016/j.cell.2013.09.034 24120142 PMC3910500

[B7] Cairns R. A. Iqbal J. Lemonnier F. Kucuk C. de Leval L. Jais J.-P. (2012). IDH2 mutations are frequent in angioimmunoblastic T-cell lymphoma. *Blood* 119 1901–1903. 10.1182/blood-2011-11-391748 22215888 PMC3293643

[B8] Dang L. White D. W. Gross S. Bennett B. D. Bittinger M. A. Driggers E. M. (2009). Cancer-associated IDH1 mutations produce 2-hydroxyglutarate. *Nature* 462 739–744. 10.1038/nature08617 19935646 PMC2818760

[B9] De Smet C. Lurquin C. Lethé B. Martelange V. Boon T. (1999). DNA methylation is the primary silencing mechanism for a set of germ line- and tumor-specific genes with a CpG-rich promoter. *Mol. Cell. Biol.* 19 7327–7335. 10.1128/MCB.19.11.7327 10523621 PMC84726

[B10] Dekker J. Marti-Renom M. A. Mirny L. A. (2013). Exploring the three-dimensional organization of genomes: interpreting chromatin interaction data. *Nat. Rev. Genet.* 14 390–403. 10.1038/nrg3454 23657480 PMC3874835

[B11] DiNardo C. D. Schimmer A. D. Yee K. W. L. Hochhaus A. Kraemer A. Carvajal R. D. (2016). A phase I study of IDH305 in patients with advanced malignancies including relapsed/refractory AML and MDS that harbor IDH1R132 mutations. *Blood* 128:1073.

[B12] Dixon J. R. Selvaraj S. Yue F. Kim A. Li Y. Shen Y. (2012). Topological domains in mammalian genomes identified by analysis of chromatin interactions. *Nature* 485 376–380. 10.1038/nature11082 22495300 PMC3356448

[B13] Dogan S. Chute D. J. Xu B. Ptashkin R. N. Chandramohan R. Casanova-Murphy J. (2017). Frequent IDH2 R172 mutations in undifferentiated and poorly-differentiated sinonasal carcinomas. *J. Pathol.* 242 400–408. 10.1002/path.4915 28493366 PMC5639875

[B14] El-Sharnouby S. Fischer B. Magbanua J. P. Umans B. Flower R. Choo S. W. (2017). Regions of very low H3K27me3 partition the *Drosophila* genome into topological domains. *PLoS One* 12:e0172725. 10.1371/journal.pone.0172725 28282436 PMC5345799

[B15] Essafi A. Webb A. Berry R. L. Slight J. Burn S. F. Spraggon L. (2011). A Wt1-controlled chromatin switching mechanism underpins tissue-specific Wnt4 activation and repression. *Dev. Cell* 21 559–574. 10.1016/J.DEVCEL.2011.07.014 21871842 PMC3604688

[B16] Flavahan W. A. Drier Y. Liau B. B. Gillespie S. M. Venteicher A. S. Stemmer-Rachamimov A. O. (2016). Insulator dysfunction and oncogene activation in IDH mutant gliomas. *Nature* 529 110–114. 10.1038/nature16490 26700815 PMC4831574

[B17] Gagné M. L. Boulay K. Topisirovic I. Huot M.É Mallette F. A. (2017). Oncogenic activities of IDH1/2 mutations: from epigenetics to cellular signaling. *Trends Cell Biol.* 27 738–752. 10.1016/j.tcb.2017.06.002 28711227

[B18] Hanssen L. L. P. Kassouf M. T. Oudelaar A. M. Biggs D. Preece C. Downes D. J. (2017). Tissue-specific CTCF–cohesin-mediated chromatin architecture delimits enhancer interactions and function in vivo. *Nat. Cell Biol.* 19 952–961. 10.1038/ncb3573 28737770 PMC5540176

[B19] Hata K. Okano M. Lei H. Li E. (2002). Dnmt3L cooperates with the Dnmt3 family of de novo DNA methyltransferases to establish maternal imprints in mice. *Development* 129 1983–1993. 11934864 10.1242/dev.129.8.1983

[B20] Jo V. Y. Chau N. G. Hornick J. L. Krane J. F. Sholl L. M. (2017). Recurrent IDH2 R172X mutations in sinonasal undifferentiated carcinoma. *Mod. Pathol.* 30 650–659. 10.1038/modpathol.2016.239 28084339

[B21] Liu X. Kato Y. Kaneko M. K. Sugawara M. Ogasawara S. Tsujimoto Y. (2013). Isocitrate dehydrogenase 2 mutation is a frequent event in osteosarcoma detected by a multi-specific monoclonal antibody MsMab-1. *Cancer Med.* 2 803–814. 10.1002/cam4.149 24403254 PMC3892385

[B22] Lu C. Ward P. S. Kapoor G. S. Rohle D. Turcan S. Abdel-Wahab O. (2012). IDH mutation impairs histone demethylation and results in a block to cell differentiation. *Nature* 483 474–478. 10.1038/nature10860 22343901 PMC3478770

[B23] Mito J. K. Bishop J. A. Sadow P. M. Stelow E. B. Faquin W. C. Mills S. E. (2018). Immunohistochemical detection and molecular characterization of IDH-mutant sinonasal undifferentiated carcinomas. *Am. J. Surg. Pathol.* 42 1067–1075. 10.1097/PAS.0000000000001064 29683816

[B24] Modrek A. S. Golub D. Khan T. Bready D. Prado J. Bowman C. (2017). Low-Grade Astrocytoma mutations in IDH1, P53, and ATRX cooperate to block differentiation [.] Materials and methods. *Cell Rep.* 21 1267–1280. 10.1016/j.celrep.2017.10.009 29091765 PMC5687844

[B25] Mondesir J. Willekens C. Touat M. de Botton S. (2016). IDH1 and IDH2 mutations as novel therapeutic targets: current perspectives. *J. Blood Med.* 7 171–180. 10.2147/JBM.S70716 27621679 PMC5015873

[B26] Murugan K. Babu K. Sundaresan R. Rajan R. Sashital D. G. (2017). The revolution continues: newly discovered systems expand the CRISPR-Cas toolkit. *Mol. Cell* 68 15–25. 10.1016/J.MOLCEL.2017.09.007 28985502 PMC5683099

[B27] Nakahashi H. Kieffer Kwon K.-R. Resch W. Vian L. Dose M. Stavreva D. (2013). A Genome-wide Map of CTCF Multivalency Redefines the CTCF Code. *Cell Rep.* 3 1678–1689. 10.1016/j.celrep.2013.04.024 23707059 PMC3770538

[B28] Noushmehr H. Weisenberger D. J. Diefes K. Phillips H. S. Pujara K. Berman B. P. (2010). Identification of a CpG Island methylator phenotype that defines a distinct subgroup of Glioma. *Cancer Cell* 17 510–522. 10.1016/j.ccr.2010.03.017 20399149 PMC2872684

[B29] Ooi S. K. T. Qiu C. Bernstein E. Li K. Jia D. Yang Z. (2007). DNMT3L connects unmethylated lysine 4 of histone H3 to de novo methylation of DNA. *Nature* 448 714–717. 10.1038/nature05987 17687327 PMC2650820

[B30] Pansuriya T. C. van Eijk R. d’Adamo P. van Ruler M. A. Kuijjer M. L. Oosting J. (2011). Somatic mosaic IDH1 and IDH2 mutations are associated with enchondroma and spindle cell hemangioma in Ollier disease and Maffucci syndrome. *Nat. Genet.* 43 1256–1261. 10.1038/ng.1004 22057234 PMC3427908

[B31] Popovici-Muller J. Lemieux R. M. Artin E. Saunders J. O. Salituro F. G. Travins J. (2018). Discovery of AG-120 (Ivosidenib): a first-in-class mutant IDH1 inhibitor for the treatment of IDH1 Mutant cancers. *ACS Med. Chem. Lett.* 9 300–305. 10.1021/acsmedchemlett.7b00421 29670690 PMC5900343

[B32] Rao S. S. P. Huntley M. H. Durand N. C. Stamenova E. K. Bochkov I. D. Robinson J. T. (2014). A 3D map of the human genome at kilobase resolution reveals principles of chromatin looping. *Cell* 159 1665–1680. 10.1016/j.cell.2014.11.021 25497547 PMC5635824

[B33] Santos-Rosa H. Schneider R. Bannister A. J. Sherriff J. Bernstein B. E. Emre N. C. T. (2002). Active genes are tri-methylated at K4 of histone H3. *Nature* 419 407–411. 10.1038/nature01080 12353038

[B34] Schwartz Y. B. Linder-Basso D. Kharchenko P. V. Tolstorukov M. Y. Kim M. Li H.-B. (2012). Nature and function of insulator protein binding sites in the *Drosophila* genome. *Genome Res.* 22 2188–2198. 10.1101/gr.138156.112 22767387 PMC3483548

[B35] Soto-Reyes E. Recillas-Targa F. (2010). Epigenetic regulation of the human p53 gene promoter by the CTCF transcription factor in transformed cell lines. *Oncogene* 29 2217–2227. 10.1038/onc.2009.509 20101205

[B36] Tahiliani M. Koh K. P. Shen Y. Pastor W. A. Bandukwala H. Brudno Y. (2009). Conversion of 5-methylcytosine to 5-hydroxymethylcytosine in mammalian DNA by MLL partner TET1. *Science* 324 930–935. 10.1126/science.1170116 19372391 PMC2715015

[B37] Tsukada Y.-I. Fang J. Erdjument-Bromage H. Warren M. E. Borchers C. H. Tempst P. (2006). Histone demethylation by a family of JmjC domain-containing proteins. *Nature* 439 811–816. 10.1038/nature04433 16362057

[B38] Turcan S. Rohle D. Goenka A. Walsh L. A. Fang F. Yilmaz E. (2012). IDH1 mutation is sufficient to establish the glioma hypermethylator phenotype. *Nature* 483 479–483. 10.1038/nature10866 22343889 PMC3351699

[B39] Van Bortle K. Ramos E. Takenaka N. Yang J. Wahi J. E. Corces V. G. (2012). *Drosophila* CTCF tandemly aligns with other insulator proteins at the borders of H3K27me3 domains. *Genome Res.* 22 2176–2187. 10.1101/gr.136788.111 22722341 PMC3483547

[B40] Verhaak R. G. Hoadley K. A. Purdom E. Wang V. Qi Y. Wilkerson M. D. (2010). Integrated genomic analysis identifies clinically relevant subtypes of glioblastoma characterized by abnormalities in PDGFRA, IDH1, EGFR, and NF1. *Cancer Cell* 17 98–110. 10.1016/j.ccr.2009.12.020 20129251 PMC2818769

[B41] Weber M. Hellmann I. Stadler M. B. Ramos L. Pääbo S. Rebhan M. (2007). Distribution, silencing potential and evolutionary impact of promoter DNA methylation in the human genome. *Nat. Genet.* 39 457–466. 10.1038/ng1990 17334365

[B42] Wellen K. E. Hatzivassiliou G. Sachdeva U. M. Bui T. V. Cross J. R. Thompson C. B. (2009). ATP-citrate lyase links cellular metabolism to histone acetylation. *Science* 324 1076–1080. 10.1126/science.1164097 19461003 PMC2746744

[B43] Weller M. Wick W. von Deimling A. (2011). Isocitrate dehydrogenase mutations: a challenge to traditional views on the genesis and malignant progression of gliomas. *Glia* 59 1200–1204. 10.1002/glia.21130 21294161

[B44] Xiao M. Yang H. Xu W. Ma S. Lin H. Zhu H. (2012). Inhibition of??-KG-dependent histone and DNA demethylases by fumarate and succinate that are accumulated in mutations of FH and SDH tumor suppressors. *Genes Dev.* 26 1326–1338. 10.1101/gad.191056.112 22677546 PMC3387660

[B45] Xu W. Yang H. Liu Y. Yang Y. Wang P. Kim S.-H. (2011). Oncometabolite 2-hydroxyglutarate is a competitive inhibitor of α-ketoglutarate-dependent dioxygenases. *Cancer Cell* 19 17–30. 10.1016/J.CCR.2010.12.014 21251613 PMC3229304

[B46] Yan H. Williams D. Jin G. Mclendon R. Rasheed B. A. Yuan W. (2009). *IDH1 and IDH2 Mutations in Gliomas.* Available at: https://www.nejm.org/doi/pdf/10.1056/NEJMoa0808710 [Accessed September 18 2018]. 10.1056/NEJMoa0808710 PMC2820383 19228619

[B47] Yen K. Konteatis Z. Sui Z. Artin E. Dang L. Straley K. (2018). “Abstract B126: AG-881, a brain penetrant, potent, pan-mutant IDH (mIDH) inhibitor for use in mIDH solid and hematologic malignancies,” in *Therapeutic Agents: Other Topics*, ed. Rai M. (Philadelphia, PA: American Association for Cancer Research).

[B48] Yen K. Travins J. Wang F. David M. D. Artin E. Straley K. (2017). AG-221, a first-in-class therapy targeting acute myeloid leukemia harboring oncogenic IDH2 mutations. *Cancer Discov.* 7 478–493. 10.1158/2159-8290.CD-16-1034 28193778

